# Bordetella bronchiseptica-Mediated Interference Prevents Influenza A Virus Replication in the Murine Nasal Cavity

**DOI:** 10.1128/spectrum.04735-22

**Published:** 2023-02-02

**Authors:** Jasmina M. Luczo, Illiassou Hamidou Soumana, Katie L. Reagin, Preston Dihle, Elodie Ghedin, Kimberly D. Klonowski, Eric T. Harvill, Stephen M. Tompkins

**Affiliations:** a Center for Vaccines and Immunology, University of Georgia, Athens, Georgia, USA; b Emory-UGA Centers of Excellence for Influenza Research and Surveillance (CEIRS), Athens, Georgia, USA; c Department of Infectious Diseases, University of Georgia, Athens, Georgia, USA; d Department of Cellular Biology, University of Georgia, Athens, Georgia, USA; e Center for Genomics and Systems Biology, New York University, New York City, New York, USA; f Laboratory of Parasitic Diseases, National Institute of Allergy and Infectious Diseases (NIAID), National Institutes of Health (NIH), Bethesda, Maryland, USA; g Center for Influenza Disease and Emergence Response (CIDER), Athens, Georgia, USA; Texas A&M University

**Keywords:** *Bordetella bronchiseptica*, dysbiosis, influenza A virus, mice, microbiota

## Abstract

Colonization resistance, also known as pathogen interference, describes the ability of a colonizing microbe to interfere with the ability of an incoming microbe to establish infection, and in the case of pathogenic organisms, cause disease in a susceptible host. Furthermore, colonization-associated dysbiosis of the commensal microbiota can alter host immunocompetence and infection outcomes. Here, we investigated the role of Bordetella bronchiseptica nasal colonization and associated disruption of the nasal microbiota on the ability of influenza A virus to establish infection in the murine upper respiratory tract. Targeted sequencing of the microbial 16S rRNA gene revealed that B. bronchiseptica colonization of the nasal cavity efficiently displaced the resident commensal microbiota—the peak of this effect occurring 7 days postcolonization—and was associated with reduced influenza associated-morbidity and enhanced recovery from influenza-associated clinical disease. Anti-influenza A virus hemagglutinin-specific humoral immune responses were not affected by B. bronchiseptica colonization, although the cellular influenza PA-specific CD8^+^ immune responses were dampened. Notably, influenza A virus replication in the nasal cavity was negated in B. bronchiseptica-colonized mice. Collectively, this work demonstrates that B. bronchiseptica-mediated pathogen interference prevents influenza A virus replication in the murine nasal cavity. This may have direct implications for controlling influenza A virus replication in, and transmission events originating from, the upper respiratory tract.

**IMPORTANCE** The interplay of microbial species in the upper respiratory tract is important for the ability of an incoming pathogen to establish and, in the case of pathogenic organisms, cause disease in a host. Here, we demonstrate that B. bronchiseptica efficiently colonizes and concurrently displaces the commensal nasal cavity microbiota, negating the ability of influenza A virus to establish infection. Furthermore, B. bronchiseptica colonization also reduced influenza-associated morbidity and enhanced recovery from influenza-associated disease. Collectively, this study indicates that B. bronchiseptica-mediated interference prevents influenza A virus replication in the upper respiratory tract. This result demonstrates the potential for respiratory pathogen-mediated interference to control replication and transmission dynamics of a clinically important respiratory pathogen like influenza A virus.

## INTRODUCTION

Human influenza A viruses (IAVs) are extremely contagious respiratory pathogens that cause annual seasonal epidemics, with an estimated attack rate of 5 to 10% of adults and 20 to 30% of children globally (1). Human IAV infection is associated with a high disease burden, particularly in the young, elderly, pregnant, those with chronic diseases, and the immunosuppressed (2), resulting in global estimates of 1 billion cases, 3 to 5 million severe cases (3), and ~290,000 to 650,000 influenza-related deaths each year (4). Human IAVs primarily target ciliated epithelial cells of the respiratory tract (5, 6) and viral shedding from nasal epithelium is thought to be directly responsible for human IAV transmission events (7).

Colonization of the respiratory tract represents a crucial step for the establishment of an infection by a respiratory pathogen (8–10). The respiratory epithelium hosts a complex microbial community, collectively termed the microbiota, that has been shown to influence host respiratory immunocompetence (11). The respiratory microbiota can also interact directly or indirectly with incoming pathogens and modulate their ability to establish an infection, a process known as colonization resistance (12). Furthermore, bacterial infection and associated dysbiosis of the respiratory microbiome can alter viral respiratory infection outcomes. For example, it has been shown that immune priming by Staphylococcus aureus ameliorates influenza-induced lung immunopathology (13) and priming of human macrophages by bacterial lipopolysaccharide inhibits influenza infection and dampens CD8^+^ T-cell responses (14). Finally, early studies exploring the pathogen interference phenomenon demonstrated that colonization of the newborn infant nasal cavity with a relatively nonpathogenic Staphylococcus aureus strain (502A) prevented colonization with pathogenic S. aureus strains and interrupted the chain of transmission (15).

Mammalian *Bordetella* species (B. pertussis, *B*. *parapertussis*, B. bronchiseptica) are genetically conserved, Gram-negative aerobic coccobacilli that cause respiratory tract infection in mammals (16). Of clinical relevance to human health is B. pertussis, the major aetiological agent of whooping cough (17). In contrast to the restricted host range of B. pertussis, B. bronchiseptica colonizes various animal hosts with symptoms ranging from asymptomatic colonization to severe pneumonia (18); however, B. bronchiseptica rarely causes clinical disease in humans (19). Like human IAVs, *Bordetella* species target ciliated epithelial cells of the respiratory tract (20–23). While adherence to, and replication of *Bordetella* species was thought to occur extracellularly (23–26), evidence for epithelial cell invasion has been reported (27, 28).

Of the mammalian *Bordetella* spp., B. bronchiseptica has the extraordinary ability to displace the murine nasal microbiota (29) and establish persistent colonization (30–32). In contrast, B. pertussis fails to overcome the murine nasal microbiota (29, 33). B. pertussis colonization has been shown to exacerbate IAV infection (34); however, very little is known about the impact of B. bronchiseptica colonization on IAV infection dynamics and disease outcomes. Interestingly, a live attenuated B. pertussis vaccine candidate protected mice from IAV-induced mortality (35). Additionally, a live attenuated influenza vaccine (LAIV) efficacy study in pigs demonstrated that B. bronchiseptica colonization of the upper respiratory tract reduced LAIV protective efficacy (36), suggesting that the ability of LAIV to replicate in the upper respiratory tract was restricted.

Given the ability of B. bronchiseptica to impact the nasal microbial community and reduce the efficacy of LAIV, we investigated the role of B. bronchiseptica nasal colonization and associated disturbance of the nasal microbiota on altering IAV infection, disease, and immune response outcomes. Dysbiosis of the nasal microbiome by B. bronchiseptica colonization was associated with reduced IAV associated-morbidity and enhanced recovery from IAV-associated clinical disease, the peak of this effect occurring 7 days postcolonization. Furthermore, IAV replication in the nasal cavity was prevented in B. bronchiseptica-colonized mice. This effect was not observed following antibiotic treatment, suggesting the effect was B. bronchiseptica specific and was not mediated by commensal microbiota. Anti-IAV hemagglutinin (HA)-specific serum antibody responses were not affected by B. bronchiseptica colonization, although the cellular influenza PA-specific CD8^+^ immune responses were dampened. Collectively, these results suggest that B. bronchiseptica reduces IAV-associated clinical disease and prevents IAV replication in the murine upper respiratory tract. This observation is likely to have direct implications for controlling IAV replication in—and transmission events originating from—the upper respiratory tract.

## RESULTS

### Bordetella bronchiseptica colonization and antibiotic treatment alters the composition of the murine nasal microbiota.

To examine changes in the nasal microbiome following colonization with B. bronchiseptica or treatment with a broad-spectrum antibiotic, 16S rRNA gene amplicon sequencing was performed on DNA extracted from the nasal cavities of B. bronchiseptica colonized, antibiotic-treated, and naive (uninfected and untreated with antibiotics) mice prior to IAV challenge (*n *= 3 to 4).

**FIG 1:**
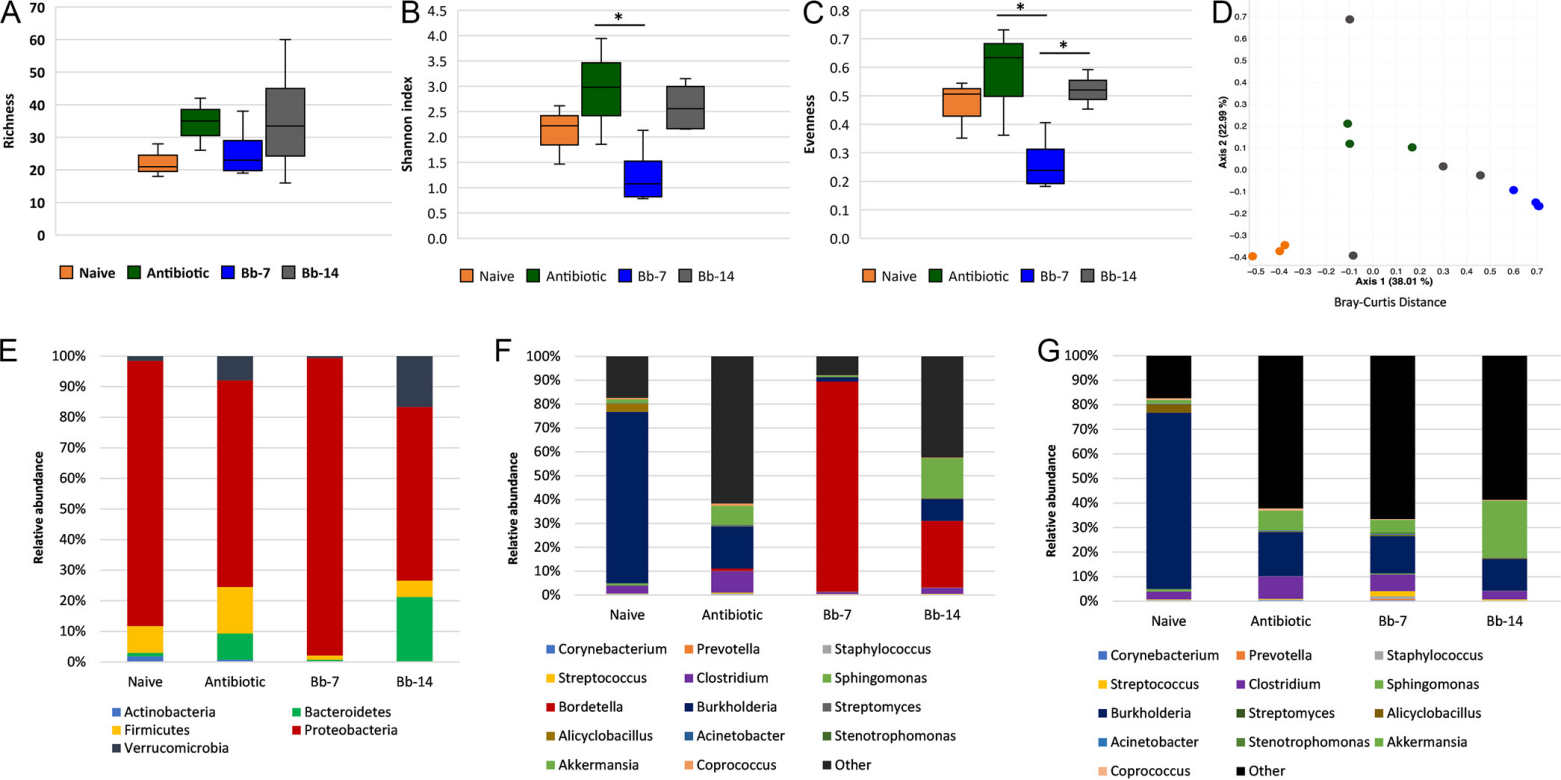
Diversity of murine nasal bacterial communities. 16S rRNA gene sequencing was performed on nasal cavities from B. bronchiseptica-colonized, antibiotic-treated, and naive mice. Microbial alpha diversity was estimated by richness (A), Shannon index (B), and evenness (C), and beta diversity estimated by Bray Curtis distance (D) with each point representing one mouse. The alpha diversity was measured for each biological replicate and is displayed per group. Differences in alpha diversity between groups were determined using a one-way ANOVA with a Tukey’s multiple-comparison test. Differences in beta diversity were estimated using PERMANOVA. Taxonomic assignments for all sequences were determined using Greengenes version 13_8. Taxa relative abundances are shown at the phylum level (E), genus level with *Bordetella* (F), or without *Bordetella* (G). *, P ≤ 0.05. (*n *= 3 to 4).

The nasal microbial richness (number of operational taxonomic units [OTU] present) varied between treatment groups, although the differences were not significant (Fig. 1A). We observed lower Shannon diversity index (a measure of taxa diversity) in the nasal microbiota of mice colonized with B. bronchiseptica 7 days prior (Bb-7) compared to naive control mice and mice colonized with B. bronchiseptica 14 days prior (Bb-14), although the difference was not statistically significant (Fig. 1B). However, we observed a significant difference in the Shannon diversity index between antibiotic-treated mice and mice colonized by B. bronchiseptica 7 days prior (Bb-7) (*P* = 0.0354) (Fig. 1B), which presented lower diversity. Additionally, mice colonized with B. bronchiseptica 7 days prior (Bb-7) were associated with decreased nasal microbial evenness (distribution of taxa abundances) compared to mice colonized with B. bronchiseptica 14 days prior (Bb-14) (*P* = 0.0164) or antibiotic-treated mice (*P* = 0.0102) (Fig. 1C).

Analysis of nasal microbial beta diversity (difference in microbial community between groups) revealed significant differences in Bray Curtis distance between naive, antibiotic-treated, and B. bronchiseptica colonized mice (permutational multivariate analysis of variance [PERMANOVA], *P* = 0.001), as visualized by a principal coordinates analysis plot (Fig. 1D).

We further analyzed the taxonomic composition of the nasal microbiota in the mice. The nasal microbial community in naive mice was composed at the phylum level by *Proteobacteria* (86%), *Firmicutes* (9%), *Actinobacteria* (2%), and *Bacteroidetes* (1%) (Fig. 1E). Antibiotic-treated mice displayed a greater relative abundance of *Firmicutes* (15%) and *Bacteroidetes* (8%) phyla and less *Proteobacteria* (68%) compared to naive mice. The *Proteobacteria* phylum dominated the bacterial community in the murine nasal cavity on days 14 (Bb-14) and 7 (Bb-7) post B. bronchiseptica colonization with an average relative abundance of 57% and 97%, respectively (Fig. 1E). The genus *Bordetella* represented the dominant taxa among the assigned genera in the nasal cavity of colonized mice, with an average relative abundance of 88% and 28% of microbial community on days 7 and 14 postinoculation with B. bronchiseptica, respectively (Fig. 1F). This observation suggests that B. bronchiseptica readily colonizes the murine nasal cavity and efficiently displaces the resident microbiota to become the dominant bacterial species. Although B. bronchiseptica colonization and associated microbiota displacement persisted at 14 days postinfection (dpi), the relative abundance of *Bordetella* was lower than its abundance on day 7. At the genus level, the nasal microbial community in antibiotic-treated mice showed greater relative abundance of *Clostridium* and *Sphingomonas* and a decrease in the relative abundance of the *Burkholderia* genus relative to untreated mice. When excluding *Bordetella* from the overall taxa, we observed that antibiotic-treated, Bb-7, and Bb-14 groups of mice showed a more similar trend in genus level composition, characterized by reduced relative abundance of *Burkholderia* and an increase of *Akkermansia* compared to naive mice. (Fig. 1G).

### B. bronchiseptica colonization reduces IAV-associated morbidity and enhances recovery from IAV infection.

**FIG 2:**
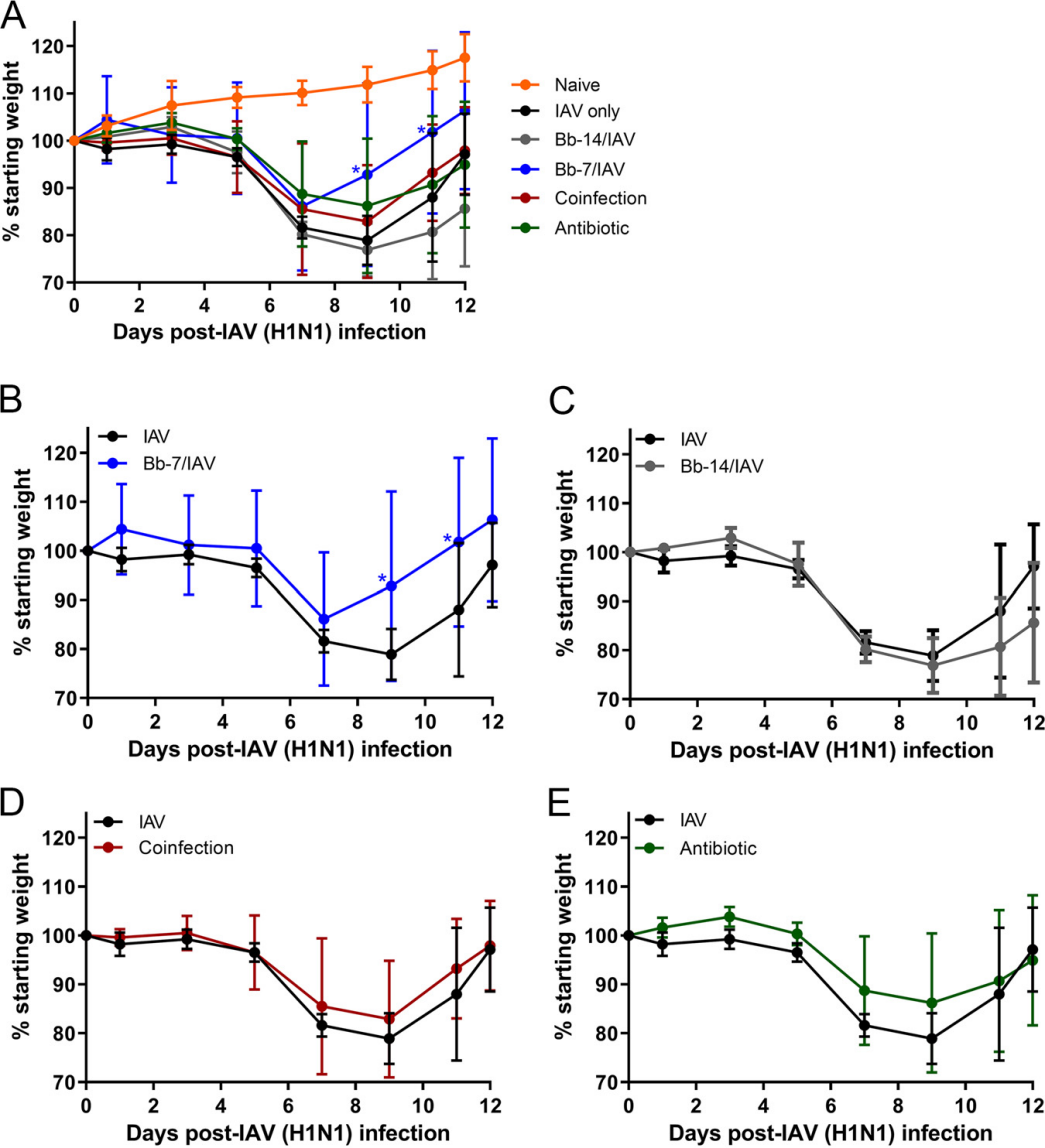
Percentage of weight change in antibiotic-treated and B. bronchiseptica-colonized mice following influenza A virus challenge. Mice colonized with B. Bronchiseptica 7 (A and B) and 14 (A and C) days prior (Bb-14 and Bb-7, respectively), antibiotic-treated (A and E), and naive mice IAV-only (A to E) were challenged with 60 PFU of A/Puerto Rico/8/1934 (H1N1) IAV in 30 μL PBS, coinfected mice (A and D) were challenged with 60 PFU of A/Puerto Rico/8/1934 (H1N1) IAV in 30 μL PBS immediately followed by 250 CFU B. Bronchiseptica RB50 in 5 μL PBS, and naive mice were sham inoculated (A) with 30 μL PBS. Weights were monitored every second day up to 12 dpi-IAV. Statistical analysis was performed using a two-way ANOVA with a Dunnett’s multiple-comparison test. All data were compared to the IAV only challenged group. *, *P* < 0.05. Error bars represent mean ± SD. *n *= 4.

To determine the effect of B. bronchiseptica colonization on IAV-associated weight loss (a measure of influenza-induced morbidity), weight change in mice challenged with IAV was monitored up to 12 days postinfection with IAV (dpi-IAV). Naive mice challenged with IAV (IAV only) induced modest weight loss at 5 dpi-IAV (3.5%) and weight loss continued up to 9 dpi-IAV, at which time IAV-only mice lost 21.1% of their starting weight. Subsequently, percentage of starting weight increased in IAV-only mice 11 dpi-IAV onwards and recovered to 97.1% of starting weight at 12 dpi-IAV (Fig. 2A to E).

Mice inoculated with B. bronchiseptica 7 days prior to IAV challenge (Bb-7/IAV) maintained percent starting weight at 5 dpi-IAV, and weight loss was not observed until 7 dpi-IAV (13.9%), which coincided with peak weight loss (Fig. 2A and B). In comparison, weight loss at 7 dpi-IAV for IAV only challenged mice was 18.4% (Fig. 2A to E). Furthermore, peak weight loss in Bb-7/IAV mice was 13.9%, a 41.1% reduction in peak weight loss compared to IAV-only mice. Subsequently, weight loss in Bb-7/IAV mice resolved from 11 dpi-IAV onwards, and mice recovered to 106.3% of starting weight at 12 dpi-IAV. Collectively, mice inoculated with B. bronchiseptica 7 days prior to IAV challenge exhibited a reduction in IAV-associated morbidity (weight loss), and IAV-associated morbidity resolved earlier than in Bb-7/IAV mice compared to the IAV only challenged group.

In contrast to results observed for Bb-7/IAV, modest weight loss in mice inoculated with B. bronchiseptica 14 days prior to IAV challenge (Bb-14/IAV) was observed at 5 dpi-IAV (2.5%) and peaked at 9 dpi-IAV (23.1%) (Fig. 2A and C), which coincided with the peak weight loss in the IAV only challenged group (21.1%). Unexpectedly, Bb-14/IAV mice recovered to only 85.6% of starting weight at 12 dpi-IAV, a 12.5% reduction compared to IAV only. Similar to Bb-14/IAV mice, weight loss in coinfected mice (Fig. 2A and D) and antibiotic-treated mice (Fig. 2A and E) also resembled that of the IAV only challenged group. Peak weight loss occurred at 9 dpi-IAV (17.1% and 13.8% for coinfected and antibiotic-treated, respectively). Coinfected and antibiotic-treated mice recovered to a percentage of starting weights comparable to that of mice challenged with IAV only.

### B. bronchiseptica modulates the ability of influenza A virus to replicate and colonize the upper respiratory tract of mice.

To examine whether B. bronchiseptica-mediated dysbiosis of the nasal microbiome influenced the replication dynamics of B. bronchiseptica or IAV in the respiratory tract, bacterial loads and virus titers were quantitated 3 and 12 days post IAV challenge (high volume inoculum, 30 μL).

**FIG 3:**
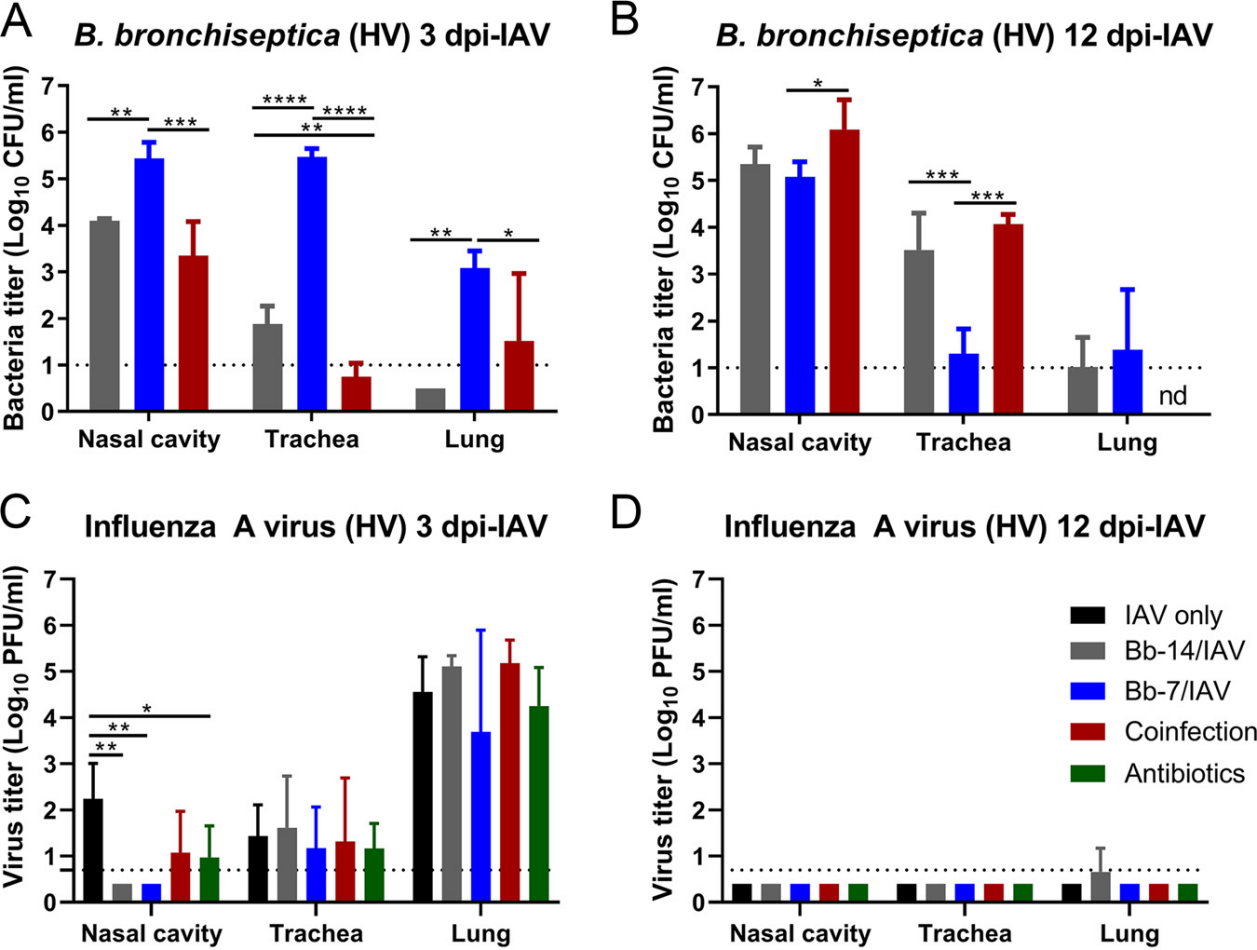
Bordetella bronchiseptica bacterial loads and influenza A virus titers detected in the murine respiratory tract following high volume inoculum influenza A virus challenge. B. bronchiseptica colonized, antibiotic-treated and naive mice were challenged with 60 PFU of A/Puerto Rico/8/1934 (H1N1) IAV in 30 μL (high volume inoculum) PBS, or coinfected with 60 PFU of A/Puerto Rico/8/1934 (H1N1) IAV in 30 μL PBS followed by 250 CFU B. Bronchiseptica RB50 in 5 μL PBS. Bacterial loads (A and B) and virus titers (C and D) were quantitated at 3 dpi-IAV (A and C) and 12 dpi-IAV (B and D) for high volume IAV inocula. Statistical analysis was performed using a one-way ANOVA with a Tukey’s (A and B) or Dunnett’s multiple-comparison test (C and D), or an unpaired *t* test (B). *, *P* < 0.05; **, *P* < 0.01; ***, *P* < 0.001; ****, *P* < 0.0001. Virus titers were compared to IAV only challenged group. Limit of detection indicated by dotted line. nd, not determined. Error bars represent mean ± SD. *n *= 4.

At 3 dpi-IAV following IAV inoculation (30 μL), B. bronchiseptica detected in Bb-7/IAV mice were significantly higher in all respiratory tissues examined (Fig. 3A). B. bronchiseptica mean loads detected in the nasal cavity, trachea, and lung of Bb-7/IAV mice at 3 dpi-IAV were 5.4, 5.5, and 3.1 log_10_ CFU/mL, respectively. In Bb-14/IAV mice, B. bronchiseptica mean loads detected at 3 dpi-IAV were several logs lower than Bb-7/IAV, with 4.1 and 1.9 log_10_ CFU/mL detected in the nasal cavity and trachea, respectively. B. bronchiseptica was not detected in the lungs of Bb-14/IAV mice. As for Bb-14/IAV mice, significantly lower levels of B. bronchiseptica were detected in coinfected mice compared to Bb-7/IAV mice at 3 dpi-IAV. Mean bacterial loads detected in coinfected mice at 3 dpi-IAV were 3.4, 0.8, and 1.5 log_10_ CFU/mL in the nasal cavity, trachea, and lung, respectively. B. bronchiseptica in the trachea of one Bb-14 mouse and the lung of two coinfected mice at 3 dpi-IAV were not quantitated due to persistent fungal growth in these samples.

At 12 dpi-IAV, B. bronchiseptica loads in the nasal cavity were similar for Bb-14/IAV mice, Bb-7/IAV mice, and coinfected mice, albeit with some variation (Fig. 3B). Mean bacteria loads in the nasal cavity at 12 dpi-IAV were 5.3, 5.1, and 6.1 log_10_ CFU/mL for Bb-14/IAV, Bb-7/IAV, and coinfected mice, respectively. In contrast, B. bronchiseptica loads in the trachea at 12 dpi-IAV were significantly lower in Bb-7/IAV mice than Bb-14/IAV or coinfected mice. Mean bacteria loads in the trachea at 12 dpi-IAV were 3.5, 1.3, and 4.1 log_10_ CFU/mL for Bb-14/IAV, Bb-7/IAV, and coinfected mice, respectively. Low levels of B. bronchiseptica were detected in the lung 12 dpi-IAV for Bb-14/IAV (mean: 1.1 log_10_ CFU/mL) and Bb-7/IAV mice (mean: 1.4 log_10_ CFU/mL). B. bronchiseptica in the lung of coinfected mice at 12 dpi-IAV were not quantitated due to persistent fungal growth in these tissue samples.

Lung virus titers detected at 3 dpi-IAV were similar in all groups following high volume IAV challenge (Fig. 3C). The presence of B. bronchiseptica had little effect on the ability of IAV to replicate in lung tissue, in agreement with a previous study (37). Mean lung virus titers for IAV-only, Bb-14/IAV, Bb-7/IAV, coinfected, and antibiotic-treated mice were 4.6, 5.1, 3.7, 5.2, and 4.3 log_10_ PFU/mL, respectively (Fig. 3C). Although the mean virus titer detected in the lung of Bb-7/IAV trended lower than other challenge groups, it was not a significant reduction. Mean virus titers detected in the trachea were similar in all challenge groups, with 1 to 2 log_10_ PFU/mL detected in all challenge groups (Fig. 3C).

In contrast, the presence of B. bronchiseptica in the nasal cavity of Bb-14/IAV and Bb-7/IAV colonized mice interfered with the ability of IAV to replicate, with viral loads being lower than the limit of detection of the assay (Fig. 3C). Coinfection of mice did not have a significant effect on viral titer detected in the nasal cavity (Fig. 3C). However, there was a modest reduction in virus titer detected in the nasal cavity of antibiotic-treated mice (Fig. 3C).

At 12 dpi-IAV, virus was not detected in the lung or trachea in any challenge group, whereas a minor amount was detected in lung tissue of only one Bb-14/IAV mouse at 12 dpi-IAV (Fig. 3D).

**FIG 4:**
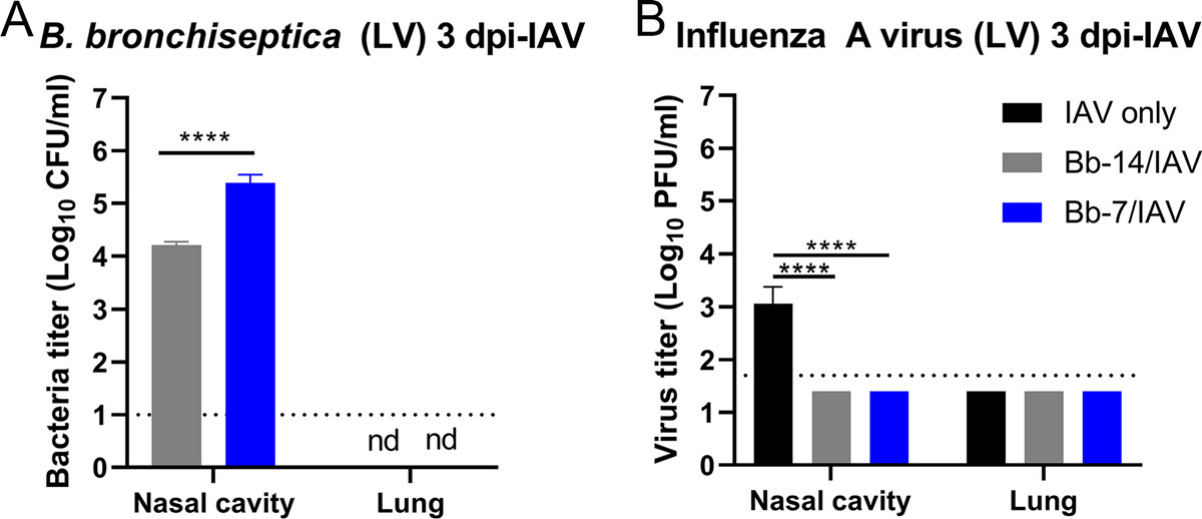
Bordetella bronchiseptica bacterial loads and influenza A virus titers detected in the murine respiratory tract following localized low volume intra nasal challenge with influenza A virus. C57BL/6 mice were colonized by B. bronchiseptica, then challenged with 60 PFU of A/Puerto Rico/8/1934 (H1N1) IAV in 5 μL (low volume inoculum) of PBS. Bacterial loads (A) and virus titers (B) were quantitated at 3 dpi-IAV. Statistical analysis was performed using unpaired *t* test (A) or one way ANOVA and Dunnett’s multiple-comparison test (B). ****, *P* < 0.0001. Virus titers are compared to IAV only challenged group. Limit of detection indicated by dotted line. nd, not determined. Error bars represent mean ± SD. *n *= 3 to 5.

To further examine and confirm the ability of B. bronchiseptica to interfere with IAV replication in the nasal cavity, Bb-14 and Bb-7-colonized mice were challenged with a low volume IAV inoculum (5 μL), which restricts virus deposition to the nasal cavity and prevents introduction of the inoculum into the lower respiratory tract (38). An inhibitor present in mouse saliva was also shown to prevent the spread of low volume intranasally instilled A/Puerto Rico/8/1934 to the lower respiratory tract (39, 40). Following low volume IAV challenge, B. bronchiseptica was detected in the nasal cavity of Bb-14/IAV and Bb-7/IAV mice at 3 dpi-IAV (Fig. 4A) at levels similar to that detected in the high volume IAV challenge group (Fig. 3A). The presence of B. bronchiseptica in the nasal cavity of Bb-14/IAV- and Bb-7/IAV-colonized mice interfered with the ability of IAV to replicate following low volume IAV challenge, with viral loads being lower than the limit of detection of the assay at 3 dpi-IAV (Fig. 4B), confirming results obtained from high volume IAV challenge. Moreover, no virus was detected in the lungs of IAV-only, Bb-14/IAV, or Bb-7/IAV mice at 3 dpi-IAV following low volume IAV challenge (Fig. 4B), confirming that low volume IAV challenge with PR8 was confined to the upper respiratory tract. Collectively, these results demonstrate that B. bronchiseptica efficiently colonizes the upper respiratory tract of mice, and the presence of B. bronchiseptica interferes with the ability of IAV to replicate in the nasal cavity.

### Colonization with B. bronchiseptica does not affect influenza A virus HA-specific antibody response; however, influenza A virus PA-specific, but not NP-specific, CD8^+^ T-cell response is altered.

**FIG 5:**
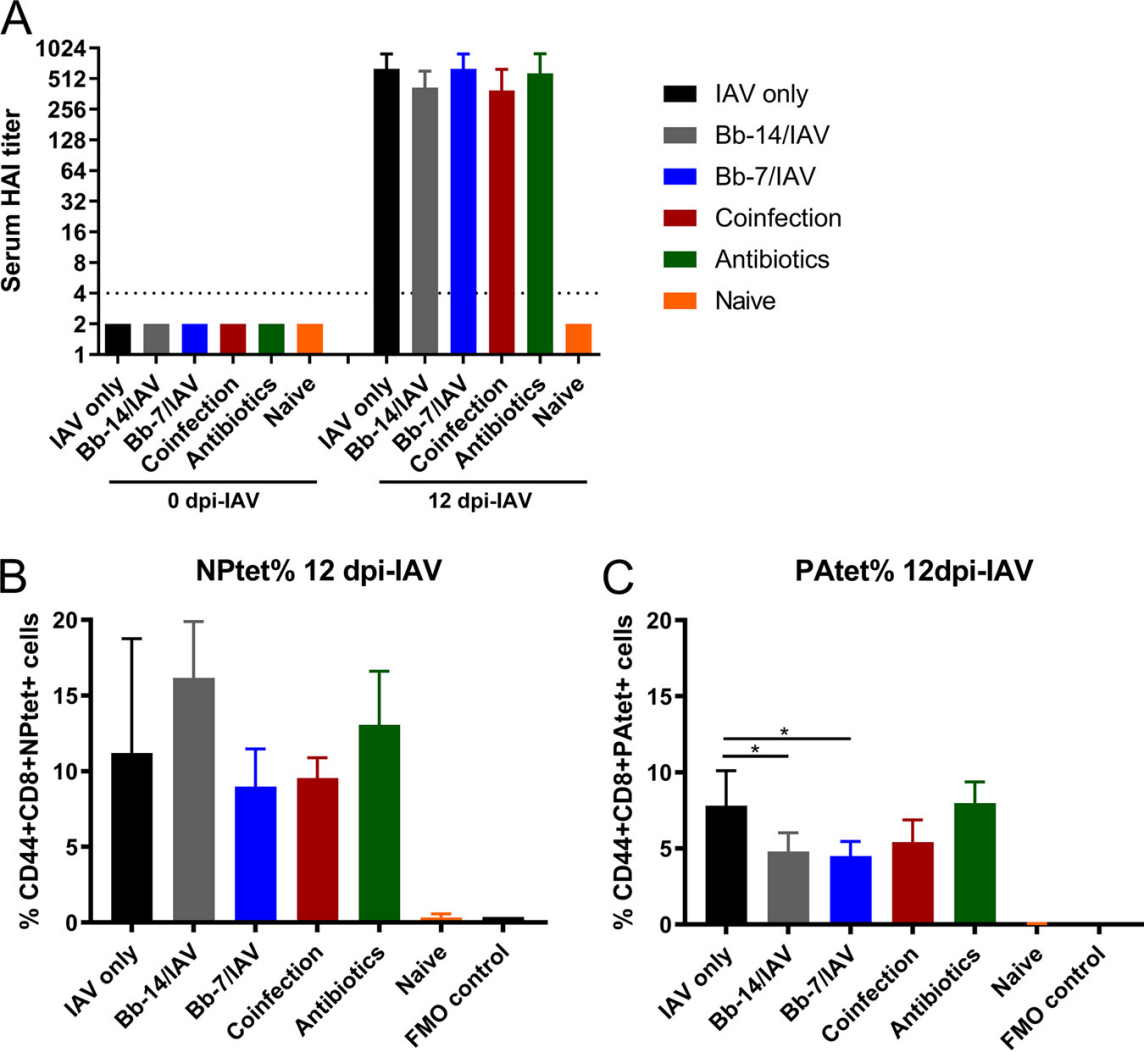
Adaptive and cellular immune responses in antibiotic-treated and B. bronchiseptica-colonized mice following influenza A virus challenge. Anti-influenza A hemagglutinin serum antibody response (A) and virus-specific CD8^+^ T-cell frequencies (B and C) were determined 12 dpi-IAV. Statistical analysis was performed using a one-way ANOVA with a Dunnett’s multiple-comparison test. *, *P* < 0.05. All data were compared to IAV only challenged group. Limit of detection indicated by dotted line. Geometric mean ± geometric SD. *n *= 4.

The influence of B. bronchiseptica colonization on the anti-IAV humoral serum antibody responses were assessed by hemagglutination inhibition (HI) assay. At 12 dpi-IAV, all mice had seroconverted and there was no significant difference in serum HI titers between challenge groups (Fig. 5A). Geometric mean HI titers detected ranged from 392 to 640 (6 to 8 log_2_).

To examine whether B. bronchiseptica colonization influenced the immunodominant influenza A virus nucleoprotein (NP)-, and polymerase acidic (PA)-specific CD8^+^ T-cell responses, CD8^+^ T cells were assessed 12 dpi-IAV challenge with high volume inoculum. Virus-specific CD44^+^CD8^+^NPtet^+^ T-cell frequencies were similar in all challenge groups (Fig. 5B). An increased frequency of CD44^+^CD8^+^NPtet^+^ T cells was detected in Bb-14/IAV mice; however, the increase was not significant. The increased frequency of CD44^+^CD8^+^NPtet^+^ T cells in Bb-14/IAV mice may be due to the delayed recovery (morbidity) from IAV infection (Fig. 2C) coupled with low levels of virus detected in the lung of this group at 12 dpi-IAV (Fig. 3D). Virus-specific CD44^+^CD8^+^NPtet^+^ T-cell frequencies in Bb-7/IAV and coinfected mice were modestly lower than the IAV-only group at 12 dpi-IAV, though this reduction was not significant. The frequency of virus-specific CD44^+^CD8^+^PAtet^+^ T cells was significantly lower in Bb-14/IAV and Bb-7/IAV mice. A modest reduction in CD44^+^CD8^+^PAtet^+^ T cells was also observed for coinfected mice; however, the reduction was not significant. Antibiotic treatment had little effect on influenza NP- and PA-specific CD8^+^ T-cell responses (Fig. 5B and C).

## DISCUSSION

Colonization of the upper respiratory tract represents a crucial step in a respiratory pathogen infectious cycle (8–10). Only pathogens that can effectively compete with commensal microorganisms present in the niche will be able to colonize, replicate, and persist (41). The microbial community living within the respiratory tract, known as the respiratory microbiome, plays a crucial role in host development, physiology, and immunity (12). Furthermore, the composition of the microbiome plays a pivotal role in respiratory health, and dysbiosis of the respiratory microbiome has been shown to alter disease outcomes following infection with bacterial and viral pathogens (12, 42–44). B. bronchiseptica has the remarkable ability to displace the murine commensal nasal microbiota (29) and to establish persistent colonization of the murine nasal cavity (30–32). While colonization with the closely related B. pertussis exacerbates IAV disease (34), little is known about B. bronchiseptica colonization and associated dysbiosis of the nasal microbiome on IAV infection outcomes. Here, we investigated the role of B. bronchiseptica nasal colonization and associated dysbiosis of the nasal microbiota on IAV replication, humoral and cellular immune responses, and disease outcomes.

It has been shown that intranasal inoculation of mice with B. bronchiseptica leads to colonization of the nasal cavity that is associated with almost complete displacement of the culturable nasal microbiota at 3 days post colonization (29). Similarly, we observed a significant reduction in microbial community diversity and a decreased relative abundance of the main genera *Firmicutes*, *Bacteroidetes*, and *Verrucomicrobia* in the nasal cavity of B. bronchiseptica-colonized mice up to day 7 postinfection, as determined by 16S rRNA target gene sequencing. Temporal analysis herein revealed that robust nasal microbiome displacement persists at 7 days post B. bronchiseptica colonization, with this effect waning by 14 days post colonization. Despite this contraction, B. bronchiseptica continued to persist in the nasal cavity at 14 days postinoculation. B. bronchiseptica persistence in the murine nasal cavity is well documented (30–32) and B. bronchiseptica has been detected up to at least 72 days post colonization (30). Interestingly, persistence in the trachea and lungs of mice is not as robust as that observed in the nasal cavity, with clearance from these organs occurring by approximately day 50 to 60 dpi (30–32). B. bronchiseptica is known to harbor an arsenal of virulence factors, allowing the bacterium to manipulate the host immune response and develop persistent colonization (30, 45–47). The clearance of B. bronchiseptica infection from the respiratory tract requires the intervention of the innate and adaptive immune responses. The closely related B. pertussis induces a significant Th1-type T-lymphocyte response characterized by increased levels of interleukin (IL)-2, interferon gamma (IFN-γ), and tumor necrosis factor alpha (TNF-α), but low levels of IL-5 and no IL-4 (48, 49), which ultimately leads to control and clearance of B. pertussis. Conversely, B. bronchiseptica directs the host immune response away from a classic Th1-response (50) toward a Th17-response (51) characterized by upregulation of IL-10 and IL-6 while suppressing TNF-α and IFN-γ expression (51), likely facilitating bacterial persistence. Whether or not long-term persistence in the nasal cavity is mediated by direct or indirect mechanisms is yet to be elucidated.

Previous studies have reported that B. bronchiseptica and IAV coinfection enhanced IAV-associated disease severity and pathological lesions in swine (36, 52, 53). Here, an increase in disease severity following coinfection was not observed, which may be due to host differences or due to pathogenic differences between bacteria or viral strains used. Notably, the B. bronchiseptica strains used in swine coinfections were swine isolates that cause atrophic rhinitis (53, 54), whereas the strain used herein, RB50, does not elicit pathological changes (55). Exacerbation of IAV-associated disease by the closely related B. pertussis (34) is negated by an attenuated B. pertussis strain BPZE1 (35) that lacks crucial B. pertussis virulence factors (56). This attenuated B. pertussis strain also elicits protection against lethal pneumococcal infection in mice (57). This highlights the potential for *Bordetella*-mediated interference to modulate the ability of incoming pathogens to establish infection and cause disease: however, consideration of bacterial genomic content is crucial to predicting protective outcomes against invading pathogens.

Modulation of host respiratory immunocompetence by commensal microbiota is well documented (58) and the microbiota has been shown to directly modulate IAV infection outcomes (11). Previously, it has been shown that combination antibiotic treatment altered the culturable microbiota in murine stool samples and dampened anti-IAV humoral and cellular immune responses. Specifically, lung IAV NP-specific CD4^+^ T-cell frequency was reduced, functional activity of CD4^+^ (whole virus-specific) and CD8^+^ (NP-specific) T cells as assessed by IFN-γ production were lower, and anti-IAV IgG, IgG2b, and IgA serum antibody levels were dampened (11). However, B. bronchiseptica colonization of swine did not alter IAV-specific IgA response following IAV vaccination (36). Herein, perturbation of the microbiome by antibiotic treatment of mice did not alter IAV disease outcomes. In contrast, B. bronchiseptica colonization and associated dysbiosis of the microbiome did elicit altered IAV-associated responses. We detected a trend toward a reduction in the frequency of IAV NP-specific CD8^+^ T cells for Bb-7/IAV, although this was not observed for the Bb-14/IAV challenge group likely due to delayed recovery and detection of virus in the lung of one mouse at 12 dpi-IAV. However, we did detect a reduction in IAV PA-specific CD8^+^ T-cell frequency in Bb-7/IAV and Bb-14/IAV mice. A trend toward reduced IAV NP- and PA-specific CD8^+^ T-cell frequencies was also observed, although this result was not significant. Finally, humoral antiinfluenza HA response was not affected by B. bronchiseptica colonization. To further examine whether modulation of IAV-associated disease outcomes was directly associated with nasal cavity colonization by B. bronchiseptica, or from dysbiosis of the microbiota, we compared taxa relative abundance at the genus level with and without *Bordetella* genera. Relative abundances between antibiotic-treated, Bb-14, and Bb-7 treatment groups were similar, suggesting that restriction of IAV replication in B. bronchiseptica colonized mice was a result of B. bronchiseptica-mediated interference, rather than from perturbation of the commensal nasal microbiota.

We have demonstrated, to our knowledge for the first time, that B. bronchiseptica colonization of the murine nasal cavity blocks the ability of IAV to establish infection in the murine nasal cavity. An earlier study by Hughes et al. (36) reported a reduction in LAIV efficacy in B. bronchiseptica colonized swine; however, LAIV postvaccination titers were not quantitated so we can only speculate that the ability of LAIV to replicate in swine nasal cavity was impeded, which lead to reduced efficacy. Neutrophils and macrophages are recruited to the B. bronchiseptica-colonized respiratory tract (59) and both innate immune subsets are crucial to limiting influenza replication (60). Moreover, B. bronchiseptica colonization elicits early delivery of dendritic cells from the nasal-associated lymph nodes (61). We hypothesize that B. bronchiseptica nasal colonization leads to residency of innate immune populations that may rapidly detect and control IAV replication in the nasal cavity. The importance of recruitment and residency of innate cell populations may also explain why coinfection does not prevent IAV nasal replication as crucial cell subsets are not present to prevent infection.

An alternate hypothesis of restriction of IAV replication in the nasal cavity is that B. bronchiseptica-mediated mucin production prevents IAV host-cell binding and entry. Epithelial changes in response to B. bronchiseptica colonization includes goblet cell enlargement and increased mucus production (62) and the closely related B. pertussis significantly upregulates the mucins, MUC4 and MUC5b (63). Mucins have been shown to be protective against IAV infection by sterically inhibiting IAV receptor binding and slowing fusion (64). IAV entry into host cells is mediated by sialic acid receptors (65, 66). Notably, B. bronchiseptica can bind nasal epithelia sialylated glycoconjugates (67), likely mediated by filamentous HA (68, 69), and binding is negated by neuraminidase treatment (67), although receptor details such as sialic acid linkage is currently unknown. Due to potential shared cellular and receptor tropism, it is possible that B. bronchiseptica directly competes with IAV for sialylated receptors on nasal epithelial cells. The contribution of epithelial cell changes elicited during *Bordetella* infection should be explored in primary human epithelial cell culture models to assess the impact on influenza virus infection. Prevention of IAV infection in the nasal cavity and subsequent viruliferous state of the host is crucial to breaking the transmission cycle of IAV, as transmission events originate from the nasal mucosa (7). Competition for host cell receptor, increased mucin production impeding host receptor binding and fusion, and encountering resident innate immune cell populations may be the mechanism leading to this observed phenomenon. Nevertheless, further investigations are needed in others to gain more insight into this important observation.

## MATERIALS AND METHODS

### Virus and bacteria strains.

Influenza A virus, A/Puerto Rico/8/1934 (H1N1), was propagated by allantoic inoculation of E9-11 embryonated chicken eggs and incubated at 37°C with humidity for 3 days. Allantoic fluid was harvested, clarified by centrifugation, and stored at –80°C. Serial dilutions were performed on virus stock of known titer to obtain virus inocula of 2.0 × 10^3^ PFU/mL (high volume inoculum, 30 μL) and 1.2 × 10^4^ PFU/mL (low volume inoculum, 5 μL).

Wild-type B. bronchiseptica strain RB50 used herein has been previously described (70). B. bronchiseptica RB50 was grown on Bordet-Gengou agar plates (Difco) supplemented with 10% defibrinated sheep blood (Hemostat) and 20 μg/mL streptomycin (Sigma) for 48h at 37°C and 5% CO_2_. To prepare B. bronchiseptica RB50 inocula, Stainer-Scholte broth was inoculated with a single colony of B. bronchiseptica RB50 and incubated at 37°C and 5% CO_2_ with shaking at 200 rpm overnight. Bacterial cells were pelleted by centrifugation and resuspended in phosphate-buffered saline (PBS) (Gibco) to an optical density at 600 nm (OD_600_) of 0.1, corresponding to 1 × 10^8^ CFU/mL. Serial dilutions in PBS were performed to obtain 5 × 10^4^ CFU/mL, and mice were inoculated with 5 μL of prepared inoculum (250 CFU).

### Cells.

Madin-Darby canine kidney cells, Atlanta line, (MDCK-ATL) (International Reagent Resource FR-926) were grown in Dulbecco’s Modified Eagle’s Medium (Gibco) supplemented with 5% fetal bovine serum (Atlanta Biologics), 2 mM GlutaMAX (Gibco), and 1× antibiotic-antimycotic (Gibco). Cells were maintained at 37°C and 5% CO_2_.

### Animal experiments.

All animal experiments were reviewed and approved by the University of Georgia Institutional Animal Care Committee (AUPs: A2016 07-006-Y3-A6 and A2016 04–019-Y3-A10) and carried out in strict accordance with the National Institutes of Health Guide for the Care and Use of Laboratory Animals. Humane euthanasia of mice strictly followed American Veterinary Medical Association guidelines.

### B. bronchiseptica colonization and antibiotic administration.

Mixed sex 4 to 6-week-old wild-type C57BL/6 mice (The Jackson Laboratory) were maintained and bred at a specific pathogen-free facility at the University of Georgia (AUP: A2016 07-006-Y3-A6). Mice were randomly assigned to treatment groups and inoculated intranasally with wild-type B. bronchiseptica strain RB50 or a broad-spectrum bactericidal antibiotic (Enrofloxacin) to disturb the nasal microbiome (*n *= 4) (Fig. S1) (AUP: 2016 04–019-Y3-A10). Briefly, all mice were lightly anesthetized using 5% isoflurane and intranasally inoculated with 250 CFU B. bronchiseptica in 5 μL PBS either 14 or 7 days prior to IAV challenge, or 10 μL of 4.5 mg/mL broad-spectrum bactericidal antibiotic (Enrofloxacin) was administered onto the external nares of mice. Antibiotic treatment was performed three times at 8 h intervals with the final antibiotic treatment performed 12 h prior to IAV challenge, as previously described (29).

At 0 dpi-IAV, a subset of naive, enrofloxacin-treated mice, or mice inoculated with B. bronchiseptica RB50 7 (Bb-7) or 14 days (Bb-14) (*n *= 3 to 4) prior were humanly euthanized and nasal cavity excised to examine the nasal microbiome (Fig. S1).

### Influenza challenge.

On the day of influenza challenge (0 dpi-IAV), mice were lightly anesthetized with isoflurane and challenged intranasally with a sublethal dose of 60 PFU of A/Puerto Rico/8/1934 (H1N1) IAV in 30 μL of PBS (high volume) (*n *= 4). A coinfected group was inoculated with 60 PFU of A/Puerto Rico/8/1934 (H1N1) IAV in 30 μL of PBS immediately followed by 250 CFU of B. bronchiseptica RB50 in 5 μL of PBS. Control mice were sham challenged with PBS diluent (Fig. S1). Mouse weights were recorded every second day throughout the IAV infection period (high volume challenge groups only). Virus titers and bacterial loads in lung, trachea, and nasal cavities were quantitated 3 and 12 days postchallenge with high volume IAV (dpi-IAV). Mouse serum antiinfluenza A virus HA, and lung anti-IAV nucleoprotein (NP) and lung anti-influenza A polymerase acidic (PA) CTL responses were determined on 12 dpi-IAV (high volume IAV challenge groups only). To confirm B. bronchiseptica*’s* ability to interfere with IAV replication in the nasal cavity, mice were inoculated with 60 PFU of A/Puerto Rico/8/1934 (H1N1) IAV in 5 μL of PBS (low volume) (*n *= 3 to 5). For the low volume IAV challenge groups, virus titers and bacteria loads were quantitated in nasal cavity and lung at 3 dpi-IAV only.

### 16S amplicon sequencing of the nasal cavity microbiota.

DNA was extracted from the nasal cavity collected from naive and antibiotic-treated mice, and mice colonized with B. bronchiseptica RB50 for 7 and 14 days (*n *= 3 to 4) (0 dpi-IAV, pre-IAV challenge) using a ZymoBIOMICS DNA miniprep kit (Zymo Research) according to the manufacturers’ instructions. Bacterial 16S rRNA gene amplicons were generated by PCR amplification of the hypervariable V4 region (71). Briefly, 4 μL of DNA and 1.6 μL of each uniquely barcoded 806RB reverse primer (5 μM) were combined with 19.4 μL master mix containing 0.8 μL forward primer 515F (10 μM), 0.5 μL dNTPs (10 mM), 5 μL 5× Q5 buffer, 0.35 μL Q5 Hot Start high-fidelity DNA polymerase (New England BioLabs Inc.), and 12.75 μL nuclease-free water. Nuclease-free water was substituted for DNA in the negative control and ZymoBIOMICS Microbial Community Standard (Zymo Research) was included as a positive control. The PCR cycling conditions were as follows: 94°C for 2 min followed by 33 cycles of 94°C for 30 s, 55°C for 30 s, and 72°C for 90 s, followed by 72°C for 10 min and 4°C hold.

PCR amplicons were purified on a Bravo automated liquid handling platform (Agilent Technologies) using 0.65× volume of AMPure XP Beads (Agencourt), washed twice with 80% ethanol and eluted in 20 μL T1/10E buffer, pH 8.0. Eluted PCR amplicons were quantified with a Quant-iT double-stranded DNA high-sensitivity assay kit (Invitrogen) according to the manufacturers’ instructions. Libraries were pooled with equal input mass and each pool was repurified with 0.65× volume of AMPure XP Beads. All pools were quantified on a HSD1000 ScreenTape with the Agilent TapeStation 4200 (Agilent Technologies) to verify sample purity. The final pool was then quantified by qPCR with the KAPA library quantification kit (KAPA Biosystems) on the Roche 480 LightCycler system and diluted to 4 nM. The final library was sequenced at 2 × 250 using a MiSeq Illumina Sequencer generating 250 bp paired end reads.

### Microbial community profiling.

Analysis of the 16S rRNA gene sequencing data were performed using QIIME 2 (72). Briefly, raw data were demultiplexed and quality filtered using the q2-demux plugin, followed by denoising with DADA2 (73). An average of 30,255 reads per sample were obtained before filtering low quality and chimeric sequences, and an average of 19,579 reads remained post filtering. Filtered amplicon sequence variants were aligned with MAFFT (74) (via q2-alignment) and used to construct a phylogeny with FastTree 2 (75) (via q2-phylogeny). Alpha diversity metrics (Richness, Shannon diversity index, and Evenness) and beta diversity metrics (Bray-Curtis distance) were computed in QIIME2 (via q2-diversity). The taxonomy was assigned to amplicon sequence variants (ASVs) using the q2-feature-classifier (76) classify-sklearn naive Bayes taxonomy classifier against the Greengenes 13_8 OTUs reference sequences (77). Singletons, low abundance taxa (frequency <10 across all samples), taxa without annotation at the phylum level, and those identified as mitochondria or chloroplast were removed from the analysis. Differences in alpha diversity across treatment groups were tested using one-way ANOVA while difference beta diversity was estimated by permutational multivariate analysis of variance (PERMANOVA) (78).

### Virus and bacteria quantitation.

Influenza A virus titers in clarified tissue homogenates were quantitated by plaque assay in MDCK-ATL cells. Briefly, growth medium was aspirated from confluent MDCK-ATL monolayers and cells were washed with PBS. Clarified tissue homogenates were titrated 10-fold in PBS, added to monolayers and absorbed for 1 h at 37°C and 5% CO_2_. Cell monolayers were overlaid with Dulbecco’s Modified Eagle’s Medium supplemented with 2 mM GlutaMAX, 1× antibiotic-antimycotic, 2 μg/mL N-tosyl-l-phenylalanine chloromethyl ketone-treated trypsin (Worthington Biochemicals) and 1.2% avicel RC-581F (FMC BioPolymer) and placed at 37°C and 5% CO_2_ for 3 days. Cell monolayers were washed with PBS and fixed with acetone (80%) and methanol (20%) solution. Virus plaques were visualized by staining with crystal violet and PFU calculated.

B. bronchiseptica in nonclarified tissue homogenates were quantitated by plating 10-fold serial dilutions on Bordet-Gengou agar supplemented with 10% defibrinated sheep blood and 20 μg/mL streptomycin. Plates were incubated for 2 days at 37°C and 5% CO_2_ and bacterial CFU enumerated.

### Quantitate lung anti-influenza A virus-specific CD8^+^ T-cell response.

**(i) Isolation of lymphocytes.** Mouse lungs were harvested 12 dpi-IAV, mechanically disrupted, and placed in Hanks’ balanced salt solution (Gibco) supplemented with 1.25 μM ethylenediaminetetraacetic acid (Sigma) and 1× antibiotic-antimycotic. Lung tissue was stirred at 37°C for 30 min at 450 rpm in siliconized flasks prior to pelleting mechanically dissociated lung tissue by gentle centrifugation. Supernatant was aspirated and tissue pellet was resuspended and enzymatically dissociated in Roswell Park Memorial Institute (RPMI)-1640 media (Gibco) supplemented with 150 U/mL collagenase (Worthington Biochemical Corporation, CLS-1), 1.25 μM magnesium chloride, 1.25 μM calcium chloride, 1× antibiotic-antimycotic, and 5% fetal bovine serum at 37°C for 60 min at 550 rpm in siliconized flasks. Tissue suspensions were passed through 40-μM cell strainers, lung tissue was pelleted by gentle centrifugation before resuspending in 44% Percoll in RPMI 1640 media overlaid on 67% Percoll in PBS. Tissue suspensions were centrifuged at 1,700 × *g* for 20 min and buffy coat was aspirated, washed with RPMI 1640 media, and lymphocytes were resuspended in PBS.

**(ii) Tetramer staining of cytotoxic T lymphocytes.** Influenza A virus nucleoprotein (NP_366_-ASNENMETM) and polymerase acidic (PA_224_-SSLENFRAYV) MHC class I tetramer (NIH Tetramer Facility) staining was conducted at room temperature for 1 h along with other surface-staining antibodies: αCD44 (53-6.7) and αCD8α (IM7) (Tonbo Biosciences). Samples were acquired on a Becton, Dickinson LSRII flow cytometer with FacsDiva Software (BD Biosciences), and data were analyzed using FlowJo software, version 10.0.8r1 (Tree Star Inc.). All samples were gated on single cells and lymphocytes prior to further gating analysis.

### Hemagglutination inhibition assay.

Mouse sera were collected 14 dpi-IAV and 12 dpi-IAV and heat inactivated at 56°C for 1 h. Heat-inactivated sera were absorbed on 10% turkey erythrocytes. Briefly, an equal volume of 10% turkey erythrocytes in PBS were added to sera and incubated at 4°C for 1 h. Sera were centrifuged at 1,000 × *g* for 5 min to pellet erythrocytes, and absorbed sera were transferred to fresh tubes. Hemagglutination inhibition assay was performed as described by the World Organization for Animal Health (79), using absorbed antisera and turkey erythrocytes. Briefly, 25 μL of absorbed antisera was diluted 2-fold in PBS and 25 μL of 4 HAU homologous antigen was added and incubated at 37°C for 30 min. Then, 0.5% turkey erythrocytes were added, and hemagglutination inhibition titers were read after 45 min.

### Statistical analyses.

Statistical analysis was performed using GraphPad Prism, version 9.2.0 (GraphPad Software, Inc., USA). Beta diversity was estimated by permutational multivariate analysis of variance in QIIME2.

### Data availability.

16S amplicon sequence data are deposited in NCBI Sequence Read Archive under accession numbers BioProject accession number PRJNA879289. The sequence data are accessible at the following URLs: https://www.ncbi.nlm.nih.gov/biosample/30802857; https://www.ncbi.nlm.nih.gov/biosample/30802858; https://www.ncbi.nlm.nih.gov/biosample/30802859; https://www.ncbi.nlm.nih.gov/biosample/30802860; https://www.ncbi.nlm.nih.gov/biosample/30802861; https://www.ncbi.nlm.nih.gov/biosample/30802862; https://www.ncbi.nlm.nih.gov/biosample/30802863; https://www.ncbi.nlm.nih.gov/biosample/30802864; https://www.ncbi.nlm.nih.gov/biosample/30802865; https://www.ncbi.nlm.nih.gov/biosample/30802866; https://www.ncbi.nlm.nih.gov/biosample/30802867; https://www.ncbi.nlm.nih.gov/biosample/30802868; https://www.ncbi.nlm.nih.gov/biosample/30802869; https://www.ncbi.nlm.nih.gov/biosample/30802870.

## References

[1] World Health Organization. 2021. Influenza. https://www.who.int/teams/health-product-and-policy-standards/standards-and-specifications/vaccines-quality/influenza#:~:text=Both%20influenza%20A%20and%20B%20viruses%20are%20important,at%205-10%25%20in%20adults%20and%2020-30%25%20in%20children. Accessed 8 July 2021.

[2] World Health Organization. 2018. Influenza (Seasonal). http://www.who.int/mediacentre/factsheets/fs211/en/. Accessed 3 July 2018.

[3] World Health Organization. 2019. Global Influenza Strategy, 2019-2030. https://www.who.int/publications/i/item/9789241515320. Accessed 8 July 2021.

[4] Iuliano AD, Roguski KM, Chang HH, Muscatello DJ, Palekar R, Tempia S, Cohen C, Gran JM, Schanzer D, Cowling BJ, Wu P, Kyncl J, Ang LW, Park M, Redlberger-Fritz M, Yu H, Espenhain L, Krishnan A, Emukule G, van Asten L, Pereira da Silva S, Aungkulanon S, Buchholz U, Widdowson M-A, Bresee JS, Azziz-Baumgartner E, Cheng P-Y, Dawood F, Foppa I, Olsen S, Haber M, Jeffers C, MacIntyre CR, Newall AT, Wood JG, Kundi M, Popow-Kraupp T, Ahmed M, Rahman M, Marinho F, Sotomayor Proschle CV, Vergara Mallegas N, Luzhao F, Sa L, Barbosa-Ramírez J, Sanchez DM, Gomez LA, Vargas XB, Acosta Herrera a, Llanés MJ, Global Seasonal Influenza-associated Mortality Collaborator Network., et al. 2018. Estimates of global seasonal influenza-associated respiratory mortality: a modelling study. Lancet 391:1285–1300. doi:10.1016/S0140-6736(17)33293-2. 29248255 PMC5935243

[5] van Riel D, Munster VJ, de Wit E, Rimmelzwaan GF, Fouchier RAM, Osterhaus A, Kuiken T. 2006. H5N1 virus attachment to lower respiratory tract. Science 312:399. doi:10.1126/science.1125548. 16556800

[6] van Riel D, den Bakker MA, Leijten LME, Chutinimitkul S, Munster VJ, de Wit E, Rimmelzwaan GF, Fouchier RAM, Osterhaus ADME, Kuiken T. 2010. Seasonal and pandemic human influenza viruses attach better to human upper respiratory tract epithelium than avian influenza viruses. Am J Pathol 176:1614–1618. doi:10.2353/ajpath.2010.090949. 20167867 PMC2843453

[7] Richard M, van den Brand JMA, Bestebroer TM, Lexmond P, de Meulder D, Fouchier RAM, Lowen AC, Herfst S. 2020. Influenza A viruses are transmitted via the air from the nasal respiratory epithelium of ferrets. Nat Commun 11:766. doi:10.1038/s41467-020-14626-0. 32034144 PMC7005743

[8] Bogaert D, de Groot R, Hermans PWM. 2004. *Streptococcus pneumoniae* colonisation: the key to pneumococcal disease. Lancet Infect Dis 4:144–154. doi:10.1016/S1473-3099(04)00938-7. 14998500

[9] Robinson J. 2004. Colonization and infection of the respiratory tract: what do we know? Paediatr Child Health 9:21–24. doi:10.1093/pch/9.1.21. 19654976 PMC2719511

[10] Liang G, Bushman FD. 2021. The human virome: assembly, composition and host interactions. Nat Rev Microbiol 19:514–527. doi:10.1038/s41579-021-00536-5. 33785903 PMC8008777

[11] Ichinohe T, Pang IK, Kumamoto Y, Peaper DR, Ho JH, Murray TS, Iwasaki A. 2011. Microbiota regulates immune defense against respiratory tract influenza A virus infection. Proc Natl Acad Sci USA 108:5354–5359. doi:10.1073/pnas.1019378108. 21402903 PMC3069176

[12] Man WH, de Steenhuijsen Piters WAA, Bogaert D. 2017. The microbiota of the respiratory tract: gatekeeper to respiratory health. Nat Rev Microbiol 15:259–270. doi:10.1038/nrmicro.2017.14. 28316330 PMC7097736

[13] Wang J, Li F, Sun R, Gao X, Wei H, Li L-J, Tian Z. 2013. Bacterial colonization dampens influenza-mediated acute lung injury via induction of M2 alveolar macrophages. Nat Commun 4:2106. doi:10.1038/ncomms3106. 23820884 PMC3715851

[14] Short KR, Vissers M, de Kleijn S, Zomer AL, Kedzierska K, Grant E, Reading PC, Hermans PWM, Ferwerda G, Diavatopoulos DA. 2014. Bacterial lipopolysaccharide inhibits influenza virus infection of human macrophages and the consequent induction of CD8+ T cell immunity. J Innate Immun 6:129–139. doi:10.1159/000353905. 23970306 PMC6741508

[15] Light IJ, Sutherland JM, Schott JE. 1965. Control of a Staphylococcal outbreak in a nursery: use of bacterial interference. JAMA 193:699–704. doi:10.1001/jama.1965.03090090005001. 14328467

[16] Diavatopoulos DA, Cummings CA, Schouls LM, Brinig MM, Relman DA, Mooi FR. 2005. *Bordetella pertussis*, the causative agent of whooping cough, evolved from a distinct, human-associated lineage of *B*. PLoS Pathog 1:e45. doi:10.1371/journal.ppat.0010045. 16389302 PMC1323478

[17] Havers F, Moro PL, Hariri S, Skoff T. 2021. Pertussis. *In* Hall E, Wodi AP, Hamborsky J, Morelli V, Schillie S (ed), Epidemiology and Prevention of Vaccine-Preventable Diseases. Centers for Disease Control and Prevention, Washington, DC., USA. https://www.cdc.gov/vaccines/pubs/pinkbook/pert.html.

[18] Goodnow RA. 1980. Biology of *Bordetella bronchiseptica*. Microbiol Rev 44:722–738. doi:10.1128/mr.44.4.722-738.1980. 7010115 PMC373201

[19] Woolfrey BF, Moody JA. 1991. Human infections associated with *Bordetella bronchiseptica*. Clin Microbiol Rev 4:243–255. doi:10.1128/CMR.4.3.243. 1889042 PMC358197

[20] Tuomanen EI, Nedelman J, Hendley JO, Hewlett EL. 1983. Species specificity of *Bordetella* adherence to human and animal ciliated respiratory epithelial cells. Infect Immun 42:692–695. doi:10.1128/iai.42.2.692-695.1983. 6315583 PMC264484

[21] Yokomizo Y, Shimizu T. 1979. Adherence of *Bordetella bronchiseptica* to swine nasal epithelial cells and its possible role in virulence. Res Vet Sci 27:15–21. doi:10.1016/S0034-5288(18)32854-6. 504806

[22] Tuomanen EI, Hendley JO. 1983. Adherence of *Bordetella pertussis* to human respiratory epithelial cells. J Infect Dis 148:125–130. doi:10.1093/infdis/148.1.125. 6309991

[23] Sekiya K, Futaesaku Y, Nakase Y. 1988. Electron microscopic observations on tracheal epithelia of mice infected with *Bordetella bronchiseptica*. Microbiol Immunol 32:461–472. doi:10.1111/j.1348-0421.1988.tb01406.x. 3173144

[24] Marks MI, Stacy T, Krous HF. 1980. Progressive cough associated with lymphocytic leukemoid reaction in an infant. J Pediatr 97:156–160. 7381636

[25] Collier AM, Peterson LP, Baseman JB. 1977. Pathogenesis of infection with *Bordetella pertussis* in hamster tracheal organ culture. J Infect Dis 136:S196–S203. doi:10.1093/infdis/136.Supplement.S196. 197174

[26] Sekiya K, Futaesaku Y, Nakase Y. 1989. Electron microscopic observations on ciliated epithelium of tracheal organ cultures infected with *Bordetella bronchiseptica*. Microbiol Immunol 33:111–121. doi:10.1111/j.1348-0421.1989.tb01503.x. 2716544

[27] Lamberti Y, Gorgojo J, Massillo C, Rodriguez ME. 2013. *Bordetella pertussis* entry into respiratory epithelial cells and intracellular survival. Pathog Dis 69:194–204. doi:10.1111/2049-632X.12072. 23893966

[28] Schipper H, Krohne GF, Gross R. 1994. Epithelial cell invasion and survival of *Bordetella bronchiseptica*. Infect Immun 62:3008–3011. doi:10.1128/iai.62.7.3008-3011.1994. 8005690 PMC302913

[29] Weyrich LS, Feaga HA, Park J, Muse SJ, Safi CY, Rolin OY, Young SE, Harvill ET. 2014. Resident microbiota affect *Bordetella pertussis* infectious dose and host specificity. J Infect Dis 209:913–921. doi:10.1093/infdis/jit597. 24227794 PMC3935476

[30] Gestal MC, Howard LK, Dewan K, Johnson HM, Barbier M, Bryant C, Soumana IH, Rivera I, Linz B, Blas-Machado U, Harvill ET. 2019. Enhancement of immune response against *Bordetella* spp. by disrupting immunomodulation. Sci Rep 9:20261. doi:10.1038/s41598-019-56652-z. 31889098 PMC6937331

[31] Barchinger SE, Zhang X, Hester SE, Rodriguez ME, Harvill ET, Ades SE. 2012. sigE facilitates the adaptation of *Bordetella bronchiseptica* to stress conditions and lethal infection in immunocompromised mice. BMC Microbiol 12:179. doi:10.1186/1471-2180-12-179. 22897969 PMC3490749

[32] Harvill ET, Preston A, Cotter PA, Allen AG, Maskell DJ, Miller JF. 2000. Multiple roles for *Bordetella* lipopolysaccharide molecules during respiratory tract infection. Infect Immun 68:6720–6728. doi:10.1128/IAI.68.12.6720-6728.2000. 11083787 PMC97772

[33] Soumana IH, Linz B, Dewan K, Sarr D, Gestal M, Howard L, Caulfield A, Rada B, Harvill E. 2021. Modeling immune evasion and vaccine limitations by targeted nasopharyngeal *Bordetella pertussis* inoculation in mice. Emerg Infect Dis 27:2107–2116. doi:10.3201/eid2708.203566. 34286682 PMC8314809

[34] Ayala VI, Teijaro JR, Farber DL, Dorsey SG, Carbonetti NH. 2011. *Bordetella pertussis* infection exacerbates influenza virus infection through pertussis toxin-mediated suppression of innate immunity. PLoS One 6:e19016. doi:10.1371/journal.pone.0019016. 21533103 PMC3080395

[35] Li R, Lim A, Phoon MC, Narasaraju T, Ng JKW, Poh WP, Sim MK, Chow VT, Locht C, Alonso S. 2010. Attenuated *Bordetella pertussis* protects against highly pathogenic influenza A viruses by dampening the cytokine storm. J Virol 84:7105–7113. doi:10.1128/JVI.02542-09. 20444902 PMC2898226

[36] Hughes HR, Brockmeier SL, Loving CL. 2018. *Bordetella bronchiseptica* colonization limits efficacy, but not immunogenicity, of live-attenuated influenza virus vaccine and enhances pathogenesis after influenza challenge. Front Immunol 9:2255. doi:10.3389/fimmu.2018.02255. 30337924 PMC6180198

[37] Jeron A, Boehme JD, Volckmar J, Gereke M, Yevsa T, Geffers R, Guzmán CA, Schreiber J, Stegemann-Koniszewski S, Bruder D. 2018. Respiratory *Bordetella bronchiseptica* carriage is associated with broad phenotypic alterations of peripheral CD4+CD25+ T cells and differentially affects immune responses to secondary non-infectious and infectious stimuli in mice. Int J Mol Sci 19:2602. doi:10.3390/ijms19092602. 30200513 PMC6165163

[38] Southam DS, Dolovich M, O'Byrne PM, Inman MD. 2002. Distribution of intranasal instillations in mice: effects of volume, time, body position, and anesthesia. Am J Physiol Lung Cell Mol Physiol 282:L833–L839. doi:10.1152/ajplung.00173.2001. 11880310

[39] Ivinson K, Deliyannis G, McNabb L, Grollo L, Gilbertson B, Jackson D, Brown LE. 2017. Salivary blockade protects the lower respiratory tract of mice from lethal influenza virus infection. J Virol 91:e00624-17. doi:10.1128/JVI.00624-17. 28446669 PMC5487578

[40] Gilbertson B, Ng WC, Crawford S, McKimm-Breschkin JL, Brown LE. 2017. Mouse saliva inhibits transit of influenza virus to the lower respiratory tract by efficiently blocking influenza virus neuraminidase activity. J Virol 91:e00145-17. doi:10.1128/JVI.00145-17. 28446666 PMC5487565

[41] Walter J, Maldonado-Gómez MX, Martínez I. 2018. To engraft or not to engraft: an ecological framework for gut microbiome modulation with live microbes. Curr Opin Biotechnol 49:129–139. doi:10.1016/j.copbio.2017.08.008. 28866242 PMC5808858

[42] Khan R, Petersen FC, Shekhar S. 2019. Commensal bacteria: an emerging player in defense against respiratory pathogens. Front Immunol 10:1203. doi:10.3389/fimmu.2019.01203. 31214175 PMC6554327

[43] Domínguez-Díaz C, García-Orozco A, Riera-Leal A, Padilla-Arellano JR, Fafutis-Morris M. 2019. Microbiota and its role on viral evasion: is it with us or against us? Front Cell Infect Microbiol 9:256. doi:10.3389/fcimb.2019.00256. 31380299 PMC6657001

[44] Libertucci J, Young VB. 2019. The role of the microbiota in infectious diseases. Nat Microbiol 4:35–45. doi:10.1038/s41564-018-0278-4. 30546094

[45] Mattoo S, Yuk MH, Huang LL, Miller JF. 2004. Regulation of type III secretion in *Bordetella*. Mol Microbiol 52:1201–1214. doi:10.1111/j.1365-2958.2004.04053.x. 15130135

[46] Weyrich LS, Rolin OY, Muse SJ, Park J, Spidale N, Kennett MJ, Hester SE, Chen C, Dudley EG, Harvill ET. 2012. A type VI secretion system encoding locus is required for *Bordetella bronchiseptica* immunomodulation and persistence *in vivo*. PLoS One 7:e45892. doi:10.1371/journal.pone.0045892. 23071529 PMC3470547

[47] Rivera I, Linz B, Dewan KK, Ma L, Rice CA, Kyle DE, Harvill ET. 2019. Conservation of ancient genetic pathways for intracellular persistence among animal pathogenic Bordetellae. Front Microbiol 10:2839. doi:10.3389/fmicb.2019.02839. 31921025 PMC6917644

[48] Mills KHG, Barnard A, Watkins J, Redhead K. 1993. Cell-mediated immunity to *Bordetella pertussis*: role of Th1 cells in bacterial clearance in a murine respiratory infection model. Infect Immun 61:399–410. doi:10.1128/iai.61.2.399-410.1993. 8423070 PMC302743

[49] Petersen JW, Ibsen PH, Hasløv K, Heron I. 1992. Proliferative responses and gamma interferon and tumor necrosis factor production by lymphocytes isolated from tracheobroncheal lymph nodes and spleen of mice aerosol infected with *Bordetella pertussis*. Infect Immun 60:4563–4570. doi:10.1128/iai.60.11.4563-4570.1992. 1398968 PMC258203

[50] Skinner JA, Pilione MR, Shen H, Harvill ET, Yuk MH. 2005. *Bordetella* type III secretion modulates dendritic cell migration resulting in immunosuppression and bacterial persistence. J Immunol 175:4647–4652. doi:10.4049/jimmunol.175.7.4647. 16177111

[51] Siciliano NA, Skinner JA, Yuk MH. 2006. *Bordetella bronchiseptica* modulates macrophage phenotype leading to the inhibition of CD4+ T cell proliferation and the initiation of a Th17 immune response. J Immunol 177:7131–7138. doi:10.4049/jimmunol.177.10.7131. 17082630

[52] Loving CL, Brockmeier SL, Vincent AL, Palmer MV, Sacco RE, Nicholson TL. 2010. Influenza virus coinfection with *Bordetella bronchiseptica* enhances bacterial colonization and host responses exacerbating pulmonary lesions. Microb Pathog 49:237–245. doi:10.1016/j.micpath.2010.06.004. 20558274

[53] Kowalczyk A, Pomorska-Mól M, Kwit K, Pejsak Z, Rachubik J, Markowska-Daniel I. 2014. Cytokine and chemokine mRNA expression profiles in BALF cells isolated from pigs single infected or co-infected with swine influenza virus and *Bordetella bronchiseptica*. Vet Microbiol 170:206–212. doi:10.1016/j.vetmic.2014.02.012. 24629899

[54] Brockmeier SL, Register KB, Magyar T, Lax AJ, Pullinger GD, Kunkle RA. 2002. Role of the dermonecrotic toxin of *Bordetella bronchiseptica* in the pathogenesis of respiratory disease in swine. Infect Immun 70:481–490. doi:10.1128/IAI.70.2.481-490.2002. 11796573 PMC127710

[55] Cotter PA, Miller JF. 1994. BvgAS-mediated signal transduction: analysis of phase-locked regulatory mutants of *Bordetella bronchiseptica* in a rabbit model. Infect Immun 62:3381–3390. doi:10.1128/iai.62.8.3381-3390.1994. 8039908 PMC302969

[56] Mielcarek N, Debrie A-S, Raze D, Bertout J, Rouanet C, Younes AB, Creusy C, Engle J, Goldman WE, Locht C. 2006. Live attenuated *B. pertussis* as a single-dose nasal vaccine against whooping cough. PLoS Pathog 2:e65. doi:10.1371/journal.ppat.0020065. 16839199 PMC1487175

[57] Belcher T, Kammoun H, Coutte L, Debrie A-S, Mielcarek N, Sirard J-C, Cauchi S, Locht C. 2022. Live attenuated *Bordetella pertussis* vaccine candidate BPZE1 transiently protects against lethal pneumococcal disease in mice. Vaccine 40:1555–1562. doi:10.1016/j.vaccine.2021.01.025. 33509692

[58] Hooper LV, Littman DR, Macpherson AJ. 2012. Interactions between the microbiota and the immune system. Science 336:1268–1273. doi:10.1126/science.1223490. 22674334 PMC4420145

[59] Nagamatsu K, Kuwae A, Konaka T, Nagai S, Yoshida S, Eguchi M, Watanabe M, Mimuro H, Koyasu S, Abe A. 2009. Bordetella evades the host immune system by inducing IL-10 through a type III effector, BopN. J Exp Med 206:3073–3088. doi:10.1084/jem.20090494. 20008527 PMC2806459

[60] Tumpey TM, García-Sastre A, Taubenberger JK, Palese P, Swayne DE, Pantin-Jackwood MJ, Schultz-Cherry S, Solórzano A, Van Rooijen N, Katz JM, Basler CF. 2005. Pathogenicity of influenza viruses with genes from the 1918 pandemic virus: functional roles of alveolar macrophages and neutrophils in limiting virus replication and mortality in mice. J Virol 79:14933–14944. doi:10.1128/JVI.79.23.14933-14944.2005. 16282492 PMC1287592

[61] Gueirard P, Ave P, Balazuc A-M, Thiberge S, Huerre M, Milon G, Guiso N. 2003. *Bordetella bronchiseptica* persists in the nasal cavities of mice and triggers early delivery of dendritic cells in the lymph nodes draining the lower and upper respiratory tract. Infect Immun 71:4137–4143. doi:10.1128/IAI.71.7.4137-4143.2003. 12819105 PMC162036

[62] Gallego C, Middleton AM, Martínez N, Romero S, Iregui C. 2013. Interaction of *Bordetella bronchiseptica* and Its lipopolysaccharide with *in vitro* culture of respiratory nasal epithelium. Vet Med Int 2013:347086. doi:10.1155/2013/347086. 23555071 PMC3608130

[63] Banus S, Pennings J, Vandebriel R, Wester P, Breit T, Mooi F, Hoebee B, Kimman T. 2007. Lung response to *Bordetella pertussis* infection in mice identified by gene-expression profiling. Immunogenetics 59:555–564. doi:10.1007/s00251-007-0227-5. 17487483 PMC1914303

[64] Delaveris CS, Webster ER, Banik SM, Boxer SG, Bertozzi CR. 2020. Membrane-tethered mucin-like polypeptides sterically inhibit binding and slow fusion kinetics of influenza A virus. Proc Natl Acad Sci USA 117:12643–12650. doi:10.1073/pnas.1921962117. 32457151 PMC7293601

[65] Luczo JM, Ronzulli SL, Tompkins SM. 2021. Influenza A virus hemagglutinin and other pathogen glycoprotein interactions with NK cell natural cytotoxicity receptors NKp46, NKp44, and NKp30. Viruses 13:156. doi:10.3390/v13020156. 33494528 PMC7911750

[66] International Committee on Taxonomy of Viruses. 2012. Family - *Orthomyxoviridae*. *In* King AMQ, Adams MJ, Carstens EB, Lefkowitz EJ (ed), Virus Taxonomy Ninth Report of the International Committee on Taxonomy of Viruses. Elsevier Inc., London, England. https://www.sciencedirect.com/science/article/pii/B9780123846846000616.

[67] Ishikawa H, Isayama Y. 1987. Evidence for sialyl glycoconjugates as receptors for *Bordetella bronchiseptica* on swine nasal mucosa. Infect Immun 55:1607–1609. doi:10.1128/iai.55.7.1607-1609.1987. 3036708 PMC260565

[68] Ishikawa H, Isayama Y. 1988. Bovine erythrocyte-agglutinin as a possible adhesin of *Bordetella bronchiseptica* responsible for binding to porcine nasal epithelium. J Med Microbiol 26:205–209. doi:10.1099/00222615-26-3-205. 3392727

[69] Cotter PA, Yuk MH, Mattoo S, Akerley BJ, Boschwitz J, Relman DA, Miller JF. 1998. Filamentous hemagglutinin of *Bordetella bronchiseptica* is required for efficient establishment of tracheal colonization. Infect Immun 66:5921–5929. doi:10.1128/IAI.66.12.5921-5929.1998. 9826374 PMC108750

[70] Buboltz AM, Nicholson TL, Weyrich LS, Harvill ET. 2009. Role of the type III secretion system in a hypervirulent lineage of Bordetella bronchiseptica. Infect Immun 77:3969–77. doi:10.1128/IAI.01362-08. 19596779 PMC2738013

[71] Caporaso JG, Lauber CL, Walters WA, Berg-Lyons D, Lozupone CA, Turnbaugh PJ, Fierer N, Knight R. 2011. Global patterns of 16S rRNA diversity at a depth of millions of sequences per sample. Proc Natl Acad Sci USA 108:4516–4522. doi:10.1073/pnas.1000080107. 20534432 PMC3063599

[72] Bolyen E, Rideout JR, Dillon MR, Bokulich NA, Abnet CC, Al-Ghalith GA, Alexander H, Alm EJ, Arumugam M, Asnicar F, Bai Y, Bisanz JE, Bittinger K, Brejnrod A, Brislawn CJ, Brown CT, Callahan BJ, Caraballo-Rodríguez AM, Chase J, Cope EK, Da Silva R, Diener C, Dorrestein PC, Douglas GM, Durall DM, Duvallet C, Edwardson CF, Ernst M, Estaki M, Fouquier J, Gauglitz JM, Gibbons SM, Gibson DL, Gonzalez A, Gorlick K, Guo J, Hillmann B, Holmes S, Holste H, Huttenhower C, Huttley GA, Janssen S, Jarmusch AK, Jiang L, Kaehler BD, Kang KB, Keefe CR, Keim P, Kelley ST, Knights D, et al. 2019. Reproducible, interactive, scalable and extensible microbiome data science using QIIME 2. Nat Biotechnol 37:852–857. doi:10.1038/s41587-019-0209-9. 31341288 PMC7015180

[73] Callahan BJ, McMurdie PJ, Rosen MJ, Han AW, Johnson AJA, Holmes SP. 2016. DADA2: high-resolution sample inference from Illumina amplicon data. Nat Methods 13:581–583. doi:10.1038/nmeth.3869. 27214047 PMC4927377

[74] Katoh K, Misawa K, Kuma K-i, Miyata T. 2002. MAFFT: a novel method for rapid multiple sequence alignment based on fast Fourier transform. Nucleic Acids Res 30:3059–3066. doi:10.1093/nar/gkf436. 12136088 PMC135756

[75] Price MN, Dehal PS, Arkin AP. 2010. FastTree 2 – approximately maximum-likelihood trees for large alignments. PLoS One 5:e9490. doi:10.1371/journal.pone.0009490. 20224823 PMC2835736

[76] Bokulich NA, Kaehler BD, Rideout JR, Dillon M, Bolyen E, Knight R, Huttley GA, Gregory Caporaso J. 2018. Optimizing taxonomic classification of marker-gene amplicon sequences with QIIME 2’s q2-feature-classifier plugin. Microbiome 6:90. doi:10.1186/s40168-018-0470-z. 29773078 PMC5956843

[77] McDonald D, Price MN, Goodrich J, Nawrocki EP, DeSantis TZ, Probst A, Andersen GL, Knight R, Hugenholtz P. 2012. An improved Greengenes taxonomy with explicit ranks for ecological and evolutionary analyses of bacteria and archaea. ISME J 6:610–618. doi:10.1038/ismej.2011.139. 22134646 PMC3280142

[78] Anderson MJ. 2001. A new method for non-parametric multivariate analysis of variance. Austral Ecol 26:32–46.

[79] World Organisation for Animal Health. 2021. Avian influenza (including infection with highly pathogenic avian influenza viruses) (version adopted in May 2021). In Manual of Diagnostic Tests and Vaccines for Terrestial Animals 2021. Paris, France. https://www.woah.org/fileadmin/Home/eng/Health_standards/tahm/3.03.04_AI.pdf.

