# Supplementary material for: Bordetella bronchiseptica-Mediated Interference Prevents Influenza A Virus Replication in the Murine Nasal Cavity
Source: Microbiol Spectr. 2023 Feb 2;11(2):e04735-22. doi: 10.1128/spectrum.04735-22 (PMC10100957; doi:10.1128/spectrum.04735-22)
Supplement: Supplemental file 1 — Fig. S1. Download spectrum.04735-22-s0001.pdf, PDF file, 0.2 MB [file spectrum.04735-22-s0001.pdf]

Supplementary Figure 1

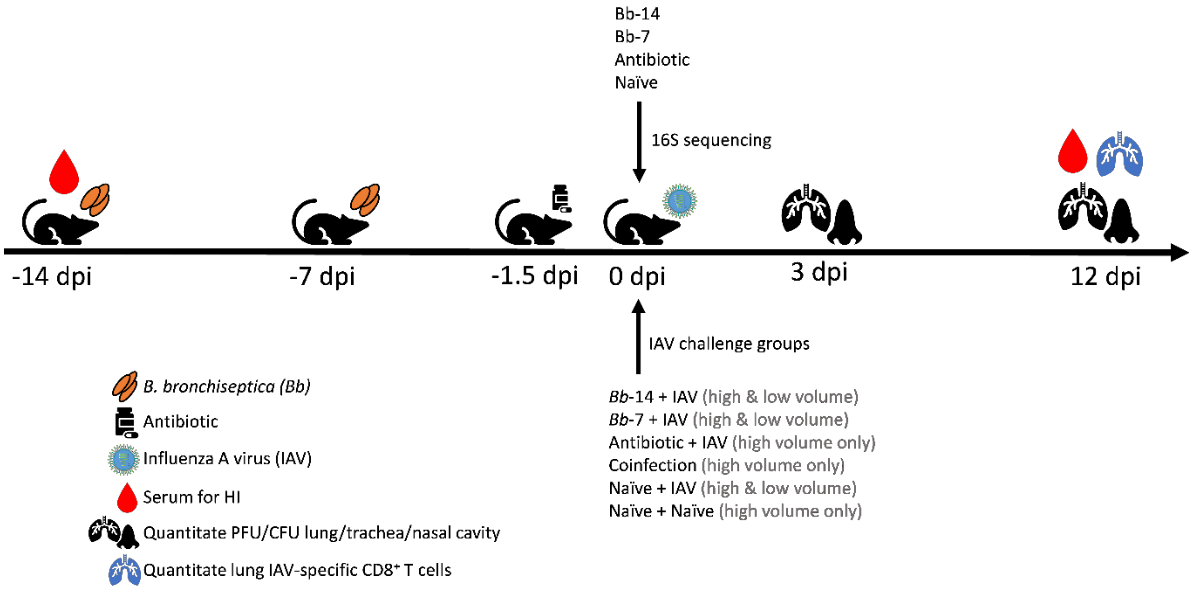

Supplementary Fig 1. Study design. Four to six-week-old C57BL/6 mice were intranasally inoculated with *B. bronchiseptica* 14 or 7 days prior to IAV challenge or treated with the broad-spectrum antibiotic, Enrofloxacin, for 1.5 days prior to influenza A challenge. Mice were challenged with high or low volume IAV inoculum. Naïve mice were sham inoculated with PBS diluent. Nasal cavity, trachea, and lungs were collected 3 and 12 dpi following high volume IAV challenge. Nasal cavity and lungs were collected 3 dpi following low volume IAV challenge. Whole blood was collected pre- and post- high volume IAV challenge.
